# Interstitial cystitis as the initial presentation of primary Sjogren’s syndrome: A case report

**DOI:** 10.1097/MD.0000000000048514

**Published:** 2026-05-15

**Authors:** Zhimin Xie, Keming Chen, Zhaochun He

**Affiliations:** a Department of Rheumatology and Immunology, The Second Affiliated Hospital of Zhejiang Chinese Medical University, Hangzhou, Zhejiang, China.

**Keywords:** autoimmune disease, bladder pain syndrome, burning micturition, interstitial cystitis, Sjögren’s syndrome

## Abstract

Interstitial Cystitis, mostly affecting middle-aged women, has been rarely associated with Sjögren’s syndrome. We report a 51-year-old woman who presented with painful micturition, pollakiuria, lower abdominal pain, and urinary urgency. In addition, the patient exhibited xerostomia, keratoconjunctivitis sicca, and leukopenia. The cystoscopy and pathological examinations confirmed the diagnosis of interstitial cystitis. Subsequent testing revealed a positive antinuclear antibody, leading to referral to the department of Rheumatology and Immunology for a labial gland biopsy, which resulted in a diagnosis of primary Sjogren’s Syndrome. This case highlights the association between Sjögren’s syndrome and interstitial cystitis, emphasizing the need for clinicians to maintain a suspicion for systemic autoimmune disorder when encountering patients with recurrent urinary tract symptoms without any other identifiable underlying cause.

## 1. Introduction

The hallmark symptoms of Sjögren’s Syndrome (SS), which is a chronic systemic autoimmune disease, include keratoconjunctivitis sicca and dry mouth, caused by lymphocytic and plasma-cell infiltration of exocrine glands, such as the salivary and lacrimal glands.^[1,2]^ The Sjögren syndrome also affects the musculoskeletal, pulmonary, renal, and nervous systems. Although the calculated prevalence of primary SS ranges from 0.1% to 3%, the true prevalence varies due to differences in diagnostic criteria, study demographics, and population demographics. The prevalence and incidence of SS vary widely, leading to inaccurate estimation of the disease trends.^[3,4]^

Bladder pain syndrome (BPS), another name for interstitial cystitis (IC), is a chronic inflammatory disease characterized by nocturia, urgency, frequency, and pelvic pain without any identifiable infection or underlying disease. The prevalence of IC/BPS ranges from 0.01% to 6.5%. The causative factor of IC/BPS is multifactorial, with proposed mechanisms including urothelial dysfunction, neurogenic inflammation, and autoimmune disorders. In China, there are only 21.8–100 cases of IC for every 100,000 people,^[5]^ which is relatively low. Studies also suggest a higher prevalence of IC among middle-aged women. Evidence suggests the presence of autoantibodies and immune complexes in patients with IC/BPS, suggesting a potential association between IC/BPS and autoimmune diseases. However, the diagnosis of IC is often delayed because it is diagnosed by exclusion.^[6,7]^ Although IC is seldom recognized as an initial manifestation of SS in literature, it has been suggested that SS has the strongest correlation with IC among all autoimmune disorders.^[8]^

We describe a 51-year-old Chinese female, presenting with IC symptoms, who was subsequently diagnosed with primary SS. This report highlights the need for considering autoimmune diseases such as SS in differential diagnosis in individuals with IC/BPS symptoms that are not improving.

## 2. Case presentation

A 51-year-old Chinese woman presented to the Department of Urology at the Second Affiliated Hospital of Zhejiang Chinese Medical Hospital with a 5-year history of chronic pelvic pain, painful micturition, urinary urgency, and pollakiuria. Her symptoms worsened with the onset of severe lower abdominal pain over the previous 2 months. The patient experienced lower abdominal pain before urination, 2 to 5 times in the night. She had no history of hematuria and had not previously sought medical care for urinary symptoms.

The patient reportedly developed sicca syndrome, including xerophthalmia and xerostomia, 3 months prior to this admission. She denied dental caries, Raynaud’s phenomenon, cough with expectoration, any rashes, joint pain, alopecia, or oral ulcers. The patient had no significant medical history, personal, marital, or childbearing history. Her parents were healthy; however, 2 of her 4 sisters had leukemia.

### 2.1. General physical examination

The patient was conscious and well-oriented with stable vitals at the time of admission. The findings of general physical examination and abdominal examination were nonsignificant.

### 2.2. Diagnostic workup

**Figure 1.**
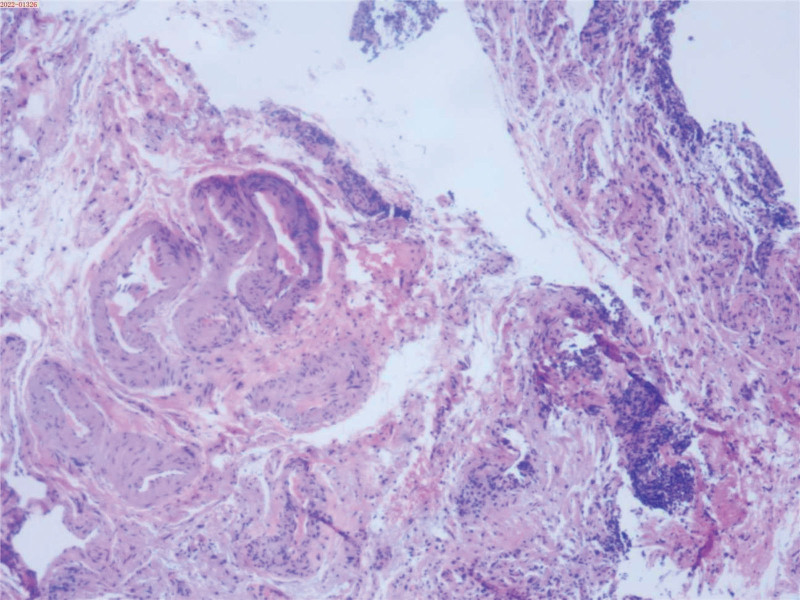
Bladder mucosa biopsy (hematoxylin and eosin staining, ×200). Histopathological examination reveals chronic mucosal inflammation and interstitial fibrous tissue hyperplasia in the trigone region, with additional acute and chronic mucosal inflammation, interstitial fibrosis, and prominent vasodilation consistent with IC/BPS.

Baseline investigations were carried out, revealing normal hemoglobin (11.9 g/L), leukopenia (WBC 2.0 × 10^9^/L), and mild thrombocytopenia (platelet count 120 × 10^9^/L). The patient’s C-reactive protein (2.91 mg/L) and erythrocyte sedimentation rate (57 mm/h) were elevated. Urinalysis was unremarkable, and urine cultures were sterile. Ultrasound KUB (kidney, ureters, and bladder) revealed no structural abnormalities. However, based on chronic lower urinary tract symptoms and unremarkable initial imaging, a provisional diagnosis of chronic cystitis was made. The patient underwent further workup, including cystoscopy, bladder hydrodistension, and transurethral laser cauterization for bladder lesions. Cystoscopy revealed extensive vascular congestion, numerous petechial hemorrhages, mucosal exfoliation, and post-dilatation changes. The histopathological examination of bladder mucosa revealed features consistent with interstitial cystitis/BPS, including chronic mucosal inflammation, interstitial fibrosis, and vasodilatation consistent with IC/BPS, *as shown in* Figure 1. The patient was diagnosed with IC/BPS based on these findings.

**Figure 2.**
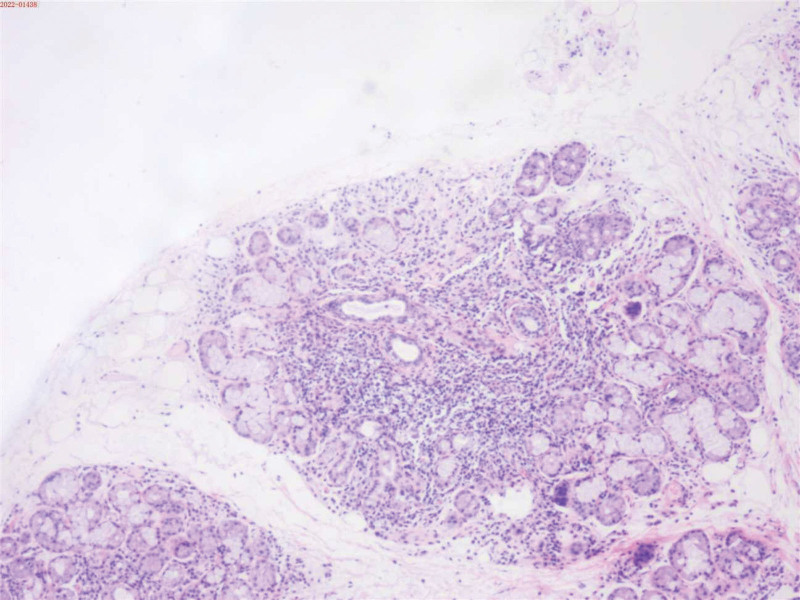
Labial salivary gland biopsy (hematoxylin and eosin staining, ×200). Histopathological examination demonstrates slight atrophy of salivary gland tissue with interstitial infiltration of lymphocytes and plasma cells, as well as multifocal lymphocytic aggregates (>50 cells per focus).

Given the sicca symptoms experienced by the patient, leukopenia, and a family history, a thorough autoimmune workup was also considered. The patient tested positive for antinuclear antibody (ANA) (1:320, speckled pattern), anti-SSA (Ro), Ro-52, and HTTP antibodies. The patient’s immunoglobulin profiling showed elevated IgG levels (19.10 g/L). Additionally, liver function test (LFTs), renal function tests (RFTs), thyroid profile, tumor markers tests, rheumatoid factor, cyclic citrullinated peptide, anticardiolipin, ANCA, hepatitis serology, and transfusion screening panels were all unremarkable. The chest computed tomography scan revealed fibrotic foci in both lungs and a pulmonary bull in the right middle lobe. Ultrasonography revealed increased echogenicity and thickening of the bilateral parotid glands; however, no obvious abnormalities were found. An ophthalmologic consultation revealed reduced tear production consistent with keratoconjunctivitis sicca. A labial gland biopsy demonstrated mild acinar atrophy along with dense lymphocytic and plasma infiltration in the interstitium. Multiple lymphocytic foci (>50 lymphocytes per 4 mm^2^) were also observed, consistent with the histopathologic criterion for Sjogren’s syndrome *as shown in* Figure 2. A definitive diagnosis of primary SS was established based on the 2016 American College of Rheumatology and European League Against Rheumatism guidelines for Sjogren’s syndrome.^[9]^ The diagnosis of SS was supported by positive SSA antibodies, Labial salivary gland’s focal lymphocytic infiltration (>1 focus/4 mm^2^), hypergammaglobulinemia, and characteristic manifestations such as dry mouth and eyes. The differential diagnoses also included systemic lupus erythematosus, sarcoidosis, lymphoma, and amyloidosis; these were excluded due to the absence of systemic manifestations, any organ-specific involvement, autoantibodies specific to lupus, or any histological evidence suggesting alternative etiologies. The patient also showed no signs of lymphoma, sarcoidosis, or amyloidosis. Thus, the patient was diagnosed with IC/BPS associated with SS.

The patient faced no diagnostic barriers because there were no financial or cultural constraints. However, due to the patient’s lack of concern towards her health, there was a delay in diagnosis, and the diagnosis was attributed to a urinary infection earlier.

### 2.3. Treatment and follow-up

The patient exhibited limited adherence to medical recommendations. The patient was advised to use artificial tears for dry eyes and was also recommended to use glucocorticoids as the first-line treatment for Sjogren syndrome. She denied using glucocorticoids and opted for hydroxychloroquine 0.1 mg twice daily. The patient declined further specialized care and was discharged from the hospital upon her request.

In a follow-up telephonic interview 6 months later, the patient reported persistent abdominal discomfort during urination and ongoing use of hydroxychloroquine at a dosage of 0.1 g twice daily. She did not report any adverse reaction to the therapy. Additional laboratory investigations performed at that time revealed (e.g., erythrocyte sedimentation rate remained elevated at 57 mm/h, C-reactive protein 2.91 mg/L, WBC 2.0 × 10^9^/L, platelets 122 × 10^9^/L), with stable renal and liver function tests. Schirmer’s test confirmed persistent dry eye, while urinalysis showed no evidence of infection. These findings suggest ongoing disease activity but stable systemic function.

## 3. Discussion

We describe a 51-year-old Chinese woman who was diagnosed with interstitial cystitis/BPS (IC/BPS) after presenting with persistent pelvic pain and urinary symptoms. After the appearance of sicca symptoms and hematological abnormalities, she was then diagnosed with primary Sjogren Syndrome (pSS). This case highlights the importance of maintaining a high suspicion for systemic autoimmune disorders in patients with refractory IC/BPS, particularly in women aged 40–60, presenting with sicca symptoms, and hematological abnormalities such as leukopenia.^[10]^

Hunner initially identified IC/BPS as a rare form of female bladder ulceration in 1915, due to its association with urinary frequency, bladder discomfort, and resistance to all conventional treatment options available at that time.^[11]^ The American Urology Association defines IC/BPS as the presence of symptoms of the lower urinary tract without infection or other recognizable causes. The characteristic symptoms include chronic pelvic pain, compression, urinary urgency, nocturia, and discomfort lasting for more than 6 weeks.^[12]^ Dry eyes and dry mouth, also referred to as Sicca syndrome, are symptoms of Primary Sjögren’s Syndrome, an autoimmune disease of the exocrine glands characterized by lymphoplasmacytic infiltration of the salivary and lacrimal glands. Additionally, it may also present with systemic features ranging from arthralgia to pulmonary and renal involvement.^[1,2]^ The fundamental pathophysiological mechanisms of SS are primarily driven by critical B-cell processes, including pro- and anti-inflammatory cytokine secretion and B-cell activation, immune complex formation, and autoantibody production. A small sample of salivary glands, usually taken from the lower lip, is important for the diagnosis of SS. In patients who are anti-Ro/La-negative, a minor salivary gland biopsy demonstrating focal lymphocytic sialadenitis (FLE) confirms SS.^[7]^

Although the estimated prevalence of IC/BPS is 6.5%, its association with systemic autoimmune diseases, including Sjogren’s syndrome, is still not well described.^[10]^ The overlap of pathological and clinical features of IC/BPS with other autoimmune disorders is suggestive of shared mechanisms of urothelial dysfunction, neurogenic inflammation, and developing autoimmunity.

Studies suggest that IC/BPS has a female preponderance with peak incidence between 40–60 years of age.^[13]^ Primary Sjogren’s syndrome also has a female-to-male ratio of 9 to 1, demonstrating female predominance, and an estimated prevalence or 0.1%–3%, with peak onset in middle age.^[14]^ Studies have explored the correlation between Sjögren’s syndrome (SS) and IC/BPS. A nationwide study found that pSS patients had IC/BPS risks that were 2.67 times greater than those of non-pSS matched controls, while women with IC/BPS had increased hazard ratios for several subsequent autoimmune disorders, such as pSS, ankylosing spondylitis, and rheumatoid arthritis, over 5 years.^[15]^

### 3.1. The pathophysiological convergence of IC/BPS and Sjogren’s syndrome

It is believed that the disruption of glycosaminoglycan (GAG), which is the layer lining the bladder epithelium, increases the bladder epithelium’s permeability to urinary solutes into the submucosal tissues, provoking the inflammatory response, such as mast cell activation, inflammatory cell recruitment, and chronic tissue injury.^[16]^ The fundamental lesions in IC/BPS found on cystoscopy are Hunner’s lesions, characterized by mucosal reddening, small blood vessels radiating toward central scars, splitting upon distention, and often accompanied by waterfall-like bleeding. Additionally, bladder petechial hemorrhage and red petechial areas that appear after hydrodistension are common. The histological examination findings are consistent with an inflammatory response to increased membrane permeability, revealing inflammatory cell infiltration, detrusor muscle hypertrophy, granulation tissue, and fibrosis within the nerve bundles.^[14]^ In pSS, lymphocytes and plasma cells infiltrate the exocrine epithelial and myoepithelium similarly, reflecting a similar vulnerability of glandular and urothelial epithelium to the body’s own immune system.

According to estimates, 25% of the general population has nonspecific autoantibodies, or ANA, which are also higher in a number of autoimmune diseases, including SS, scleroderma, and systemic lupus erythematosus.^[8]^ A significant increase in the prevalence of ANA in patients with IC/BPS has contributed to the development of an autoimmune hypothesis regarding its pathophysiology.^[17]^ Other similarities have also been noted in the immunoglobulin and complement deposits found in the bladder in the patient with autoimmune disorders with urinary symptoms, as well as in patients with IC/BPS.^[16]^

Muscarinic acetylcholine receptor subtype M3 (M3R) is distributed across salivary glands, lacrimal glands, sweat glands, bladder, and blood vessels. Anti-M_3_R antibodies in pSS patients cause cholinergic receptor-mediated glandular hypofunction. Moreover, many patients with autoantibodies directed against the M_3_R showed symptoms of bladder irritability and features suggestive of autonomic dysfunction.^[7]^ According to a nationwide study conducted in 2019, anti-M3 receptor IgG may play a significant role in the pathophysiology of IC/BPS because anti-M3R antibodies increase the expression of M3 receptors, which in turn causes detrusor muscle cells to release more inflammatory cytokines.^[15]^

Interleukin-6 (IL-6) is a crucial cytokine involved in B-cell activation, while tumor necrosis factor-alpha (TNF-α) is a significant pro-inflammatory factor. Research utilizing single-cell transcriptome sequencing has unveiled the substantial roles of IL-6 and TNF-α in the pathogenesis of IC/BPS.^[9]^ This observation may elucidate the heightened occurrence of B, as well as plasma cells in the bladder wall of individuals with IC/BPS.^[18]^ Furthermore, elevated levels of IL-6 and TNF-α have been identified in the blood of patients with SS and are associated with elevated inflammatory markers in SS.^[19]^

Local bladder inflammation results from the release of neurotransmitters by peripheral neurons in IC/BPS patients, which causes mast cells to degranulate and release pro-inflammatory mediators like histamine, serotonin, and TNF. The afferent neurons develop a positive feedback loop that increases neuropeptide release, intensifying the inflammatory responses and mast cell degranulation.^[20]^ An increase in mast cells in the salivary glands is seen in the progression of SS-related sialadenitis, and the cells produce tissue transforming growth factor β1, which promotes tissue fibrosis.^[18]^ The convergence on these common pathways is indicative of the use of systemic immunomodulatory therapies for the treatment of both glandular and bladder manifestations.

A major limitation of this case is that follow-up information was restricted to a telephonic interview, as the patient declined further in-person evaluations and additional diagnostic testing. This limited our ability to document objective progression, treatment response, or potential complications over time.

In the present case, testing for anti-M3R antibodies was not performed. Given their increasingly recognized role in both glandular and bladder dysfunction in pSS, future evaluation of such patients would benefit from assessing anti-M3R autoantibodies. This could help clarify the link between muscarinic receptor autoimmunity and bladder irritability in SS-related IC/BPS and provide a more complete picture of disease mechanisms.

### 3.2. Diagnosis and treatment modalities of SS-related IC/BPS

The IC/BPS diagnosis is still exclusionary. Consequently, it is frequently delayed by mistakenly attributing it to pelvic floor dysfunction, an overactive bladder, or recurrent UTIs. As our patient was under suspicion for chronic cystitis for 5 years before cystoscopy examination revealed characteristic bladder changes. Histopathology of the bladder mucosa confirms the diagnosis of IC/BPS.

Current guidelines also do not specify any treatment for SS-related IC/BPS; instead, they rely primarily on empirical treatment. Several strategies are used to treat IC/BPS, depending on the patient’s history and the severity of the condition.^[8]^ This may involve the use of steroids in combination with immunosuppressants such as methotrexate, cyclophosphamide, and mycophenolate mofetil, which typically improves the patient’s symptoms.^[21]^ Currently, no definitive cure is available, and the treatment is symptomatic to enhance their quality of life. Patients and their doctors must consult to decide on the best course of action. In this case, the patient declined steroids and opted for hydroxychloroquine, with limited adherence. In the advanced stages of IC/BPS, recurrent bladder inflammation may lead to bladder spasms and decreased capacity, and ultimately impact the quality of life. Therefore, when patients with SS present with evident urinary tract symptoms but no signs of urethral infection, the potential coexistence of IC/BPS should be considered to prevent misdiagnosis or oversight, as IC/BPS is diagnosed late after excluding other causes of the patient’s urinary symptoms.

## 4. Conclusion

We describe a 51-year-old woman who came in with chronic pain and refractory urinary symptoms, and was diagnosed with IC/BPS, followed by a Primary SS diagnosis. The diagnosis of IC/BPS preceded the diagnosis of Sjogren’s syndrome, which highlights the importance of considering the systemic autoimmune disorders in patients with refractory IC/BPS. The cystoscopy and bladder’s mucosa histological examination, and minor salivary glands collectively met the diagnostic criteria. Immunosuppressants and immunomodulators remain the mainstay of treatment. Although not life-threatening, SS-associated IC/BPS significantly affects quality of life. Therefore, a multi-disciplinary team for the patient’s assessment, including a urologist, rheumatologist, and ophthalmologist, is required for timely diagnosis and treatment.

Early recognition of autoimmune etiologies in patients with chronic urinary symptoms also supports Sustainable Development Goal 3 (Good Health and Well-Being) by promoting timely diagnosis, reducing morbidity, and improving overall quality of care.

## Acknowledgments

We appreciate the English language editing provided by Editage (https://www.editage.cn/).

## Author contributions

**Validation:** Zhaochun He.

**Writing – original draft:** Zhimin Xie, Keming Chen, Zhaochun He.
